# From Structural Variation of Gene Molecules to Chromatin Dynamics and Transcriptional Bursting

**DOI:** 10.3390/genes6030469

**Published:** 2015-06-30

**Authors:** Hinrich Boeger, Robert Shelansky, Heta Patel, Christopher R. Brown

**Affiliations:** Department of Molecular, Cell and Developmental Biology, University of California, Santa Cruz, CA 95064, USA; E-Mails: rshelans@ucsc.edu (R.S.); hppatel@ucsc.edu (H.P.); crb2012@gmail.com (C.R.B.)

**Keywords:** chromatin dynamics, transcriptional bursting, gene expression noise, stochastic process

## Abstract

Transcriptional activation of eukaryotic genes is accompanied, in general, by a change in the sensitivity of promoter chromatin to endonucleases. The structural basis of this alteration has remained elusive for decades; but the change has been viewed as a transformation of one structure into another, from “closed” to “open” chromatin. In contradistinction to this static and deterministic view of the problem, a dynamical and probabilistic theory of promoter chromatin has emerged as its solution. This theory, which we review here, explains observed variation in promoter chromatin structure at the level of single gene molecules and provides a molecular basis for random bursting in transcription—the conjecture that promoters stochastically transition between transcriptionally conducive and inconducive states. The mechanism of transcriptional regulation may be understood only in probabilistic terms.

## 1. Introduction

“There are great solutions, but a final solution does not exist.” (Karl Popper, 1994)

Thirty years ago, it was discovered upon mild digestion of chromatin with DNase I that transcriptional activation of the mouse mammary tumor virus coincided with a marked increase in the endonucleolytic sensitivity of viral promoter sequences [1]. Similar observations of inducible chromatin changes were subsequently made for many other genes [2]. A classic example is the *PHO5* promoter of budding yeast [3]. The observed changes were generally attributed to a transformation between qualitatively distinct chromatin structures or states. Promoter chromatin was thought to be “closed” under repressing conditions and “open” when induced for transcription. Recent research on the *PHO5* promoter suggests that this static and deterministic view is mistaken. Rather, both activated and repressed promoter chromatin are dynamical systems, describable as stochastic processes that differ only in the numerical values of their probabilistic parameters, and not the range of structural states attainable by the promoter [4]. The transition between repressed and active promoter chromatin is a change in quantity (kinetic parameter values), and not quality (structure).

**Figure 1:**
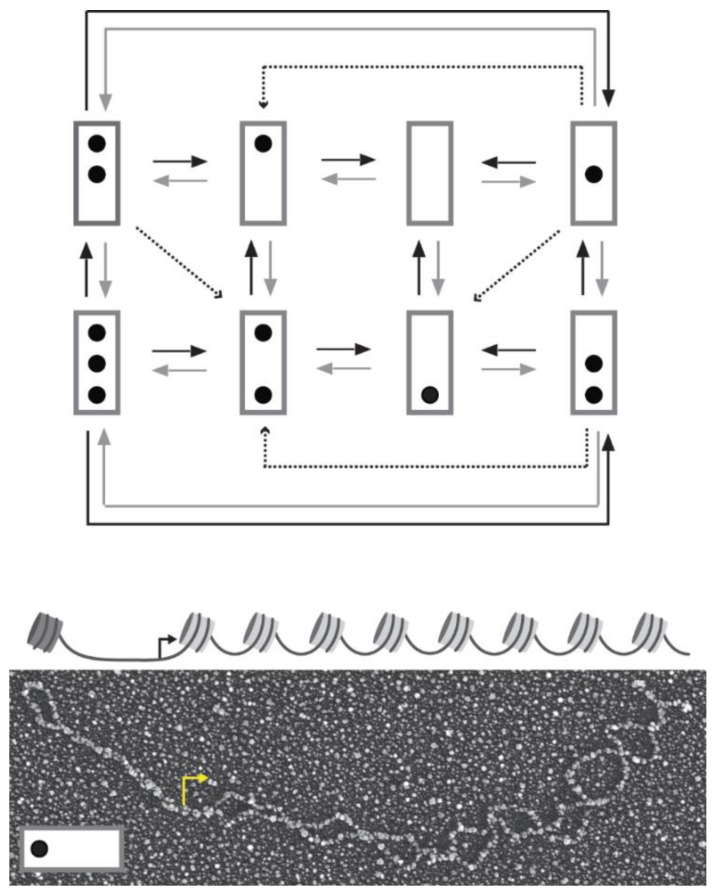
Transition graph for the stochastic dynamics of *PHO*5 promoter nucleosomes (top). The promoter is represented by a box and occupied nucleosome positions as dots. Nucleosome positions from top to bottom are called N-1 to N-3. N-1 contains the transcription start site. Black, gray, and dashed arrows indicate assembly, disassembly, and sliding transitions, respectively. At the bottom is the electron micrograph of an isolated *PHO5* molecule after psoralen-crosslinking, DNA denaturation, and heavy metal shadowing. Positions previously occupied by nucleosomes appear as single-stranded DNA “bubbles” (for psoralen crosslinks linker DNA, but not DNA within the nucleosome core particle). Nucleosome position N-3 is occupied, but not positions N-2 and N-1 (see reconstruction above electron micrograph with promoter nucleosomes in dark gray and nucleosomes over open reading frame in light gray) [4]. A bent arrow indicates the transcription start site.

Analysis of single *PHO5* gene molecules by electron microscopy revealed the existence of alternative promoter nucleosome configurations in cells that expressed *PHO5* constitutively [4]. This observation had been anticipated theoretically [5,6,7]. However, contrary to prior expectation [5], all combinatorial possibilities of occupying three nucleosome positions were observed, including the fully nucleosomal and the nucleosome-free promoter (Figure 1). The same range of configurations was observed in cells that could not activate *PHO5*, but with different relative frequencies; the fully nucleosomal configuration was, by far, the most prominent under repressing conditions, whereas a comparatively small fraction of gene molecules retained all promoter nucleosomes under activating conditions [4].

How can this structural variation be explained? In the following, we review the basic assumptions of such an explanation, its experimental tests by analysis of single gene molecules, and then discuss its implications for gene expression and regulation. Section 3 covers some formal assumptions which underlie most stochastic gene expression models, but often go without mention. A major implication of this theory is transcriptional bursting––the notion that promoters randomly transition between transcriptionally conducive and inconducive states. Excellent reviews of the rich literature on transcriptional bursting have been recently published elsewhere [8,9].

## 2. Ergodic Hypothesis

Two classes of explanatory theories for the molecular variation of promoter chromatin may be distinguished, *viz*. static and dynamical hypotheses. Different promoter nucleosome configurations may correspond, for instance, to distinct states of cellular differentiation with no transitions between cellular states and thus nucleosomal configurations [10]. This static hypothesis has been refuted: nearly all possible pairs of nucleosome configurations have been observed for two *PHO5* promoter copies that shared the same cellular history (“conjugate reporter approach”); the two copies were found to be stochastically independent [11].

It is conceivable that the promoter randomly “freezes” in distinct nucleosome configurations, regardless of the state of its (intracellular) environment. However, such an assumption neither explains variation, nor does it seem physiologically reasonable. This leaves the following alternative explanation: single gene molecules visit each promoter nucleosome configuration over time, in some sequence (see below), with statistically distributed sojourn times in between transitions [12]; in other words, the variation across a population of molecules arises from the stochastic dynamical behavior of each single molecule. We shall refer to this conjecture as “ergodic hypothesis”. (The term is also used in statistical thermodynamics with a similar but more precise meaning, but this is of no concern here.) 

## 3. Assumption of a Stationary Markov Process

Probabilistic theories make quantitative rather than qualitative predictions. Therefore, they have to be cast in mathematical form to be testable. Formal theories generally require seemingly artificial assumptions for mathematical convenience [13]. As with any other hypothesis, such assumptions are tested for their ability to provide reasonable approximations to the facts. A formalism which has proved surprisingly successful in this regard is the assumption of a time-homogeneous Markov process [13,14]––that the future is conditionally independent of the past, provided the present state is known. In short, it is assumed that the process is “memory-free”.

This lack of memory assumption is formalized as follows. The nucleosome configurations of the promoter are represented as nodes with directed edges (arrows) between them to indicate allowed transitions between configurations (Figure 1). We call the set of nodes and directed edges a “transition graph” [12]. A stochastic process may be viewed as probability mass flowing between the nodes along the edges of the graph. This flow is described by the *transition functions* of the process *p_ji_*(*t* + *h*, *t*), which indicate the probability of nucleosome configuration *j* at time *t* + *h*, given the promoter’s present configuration, *i*, at time *t*. The transition functions of a time-homogeneous Markov process depend on the time interval *h* and the present value *i* alone, and not on *t* or the process’ history prior to *t;* that is to say, *p_ji_*(*t* + *h*, *t*) = *p_ji_*(*h*, 0) ≡ *p_ji_*(*h*). This is a strong assumption; indeed, strong enough to completely specify the mathematical properties of the process [15]. Perhaps the most important of its properties is linearity: The flow of probability per unit time from node *i* to node *j* linearly depends on the probability of *i* (analogous to elementary rate equations in chemistry, but with probability replacing concentration [16]). The rate constant in this linear relationship, *i.e.*, the limit of *p_ji_*(*h*)/*h* for *h*→0, exists for all *i* ≠ *j* [15]. If there is no outgoing edge from *i* to *j*, this limit is zero. In principle, therefore, the stochastic process has as many rate constants or “kinetic parameters” as there are edges in its transition graph. Another salient property of time-homogeneous Markov processes should be mentioned here: the sojourn times between transitions are exponentially distributed.

One additional assumption is required to make testable predictions: the assumption of a stationary process. Note that a directed graph is called strongly connected if every node can be reached from any other node by a string of one or more edges. It can be proved that a time-homogeneous Markov process on a strongly connected graph tends toward a steady state where the probability currents into and out of each node are balanced; the probability at each node, then, is time-independent and uniquely defined and the process is said to be stationary [15,17]. This assumption may be experimentally satisfied, at least in good approximation [4].

## 4. Simple Process Assumption

A “good” explanation (theory) is a prohibition––it prohibits certain things to happen––or else it cannot be refuted by experimental observation [18]. The refutability of mathematical models, e.g., a stochastic process, decreases with the number of freely adjustable model parameters (degrees of freedom). Therefore, explanations with fewer degrees of freedom must be preferred over those with more [12].

The following assumption of a “simple process” dramatically limits the degrees of freedom. The assumption is this: the kinetic parameter for the transition between two nucleosome configurations only depends on the “kind of transition” [4,12]. We may distinguish between three different kinds of transitions: assembly, disassembly, and sliding. Accordingly, a transition either adds or removes nucleosomes, or redistributes nucleosomes between promoter positions. This conjecture implies that the process is a time-homogenous Markov process and it limits the degrees of freedom to two––one of the three kinetic parameter values may be set to 1 on some suitable time scale (say, the parameter for nucleosome assembly).

The simple process assumption reduces the problem of finding an explanatory theory for the relative frequencies of the promoter nucleosome configurations to the comparative analysis of alternative transition graphs. The parameter values for each graph are chosen to maximize the probability of the data. Likewise, between competing transition graphs, the graph that is preferred is that which allows the experimental observations to attain greater probability. Dynamical conjectures, *i.e.*, transition graphs, may thus be refuted given an alternative conjecture of greater likelihood [4,12]. Graphs that are not strongly connected may be excluded without calculation, because for such graphs only a subset of the experimentally observed configurations would have a probability larger than zero at steady state.

## 5. Promoter Chromatin Dynamics

It may be argued that violations of the simple process conjecture are quite plausible, that the simple process conjecture is too prohibitive or too bold. Yet, remarkably, a simple stationary process was discovered whose theoretical predictions closely correspond to microscopic observations, both for molecules isolated from induced and non-induced cells, albeit with different kinetic parameter values [4].

The transition graph of this process has the following features (Figure 1): each node of the graph has several outgoing edges, *i.e.*, the graph is “branched” [12]. Thus, single gene molecules visit the alternative promoter nucleosome configurations in random sequence. This does not imply, however, that all sequences are allowed; only sequences consistent with the transition graph are possible: nucleosomes are added, removed (by disassembly), or slid only one at a time. Sliding occurs from the central position into the outer positions of the promoter, but not *vice versa*, which explains why this position is more likely to be nucleosome-free than its neighbors. Unidirectional sliding contravenes the demand for detailed balance in thermodynamic equilibrium [19]. It follows that the steady state requires the constant input of free energy, *i.e.*, promoter chromatin is a dissipative system.

Earlier findings corroborated various aspects of this theory. The removal of nucleosomes from the *PHO5* promoter occurs by disassembly, rather than sliding away from the promoter [20]. All known remodeling enzymes accommodate one and only one nucleosome at a time [21,22,23], and they couple nucleosome transactions to ATP hydrolysis [24]. Removal of nucleosomes from the *PHO5* promoter can be largely dependent on the chromatin remodeling complex SWI/SNF [25]. As predicted by the assumption of a dynamical system of nucleosome removal and reformation, newly synthesized histones preferentially associate with promoter regions of both active and repressed promoters [26].

Our theory implies that ATP hydrolysis drives the process away from (thermodynamic) equilibrium rather than accelerates its approach toward equilibrium. This is a testable assertion: digestion of chromatin with micrococcal nuclease has suggested the presence of a “nucleosome-free region” at many promoters, in a location similar to the central (N-2) nucleosome position of the *PHO5* promoter. The free energy of nucleosome formation on the DNA of such nucleosome-free regions was found to hardly differ from a high affinity sequence (differences did not exceed one *k_B_T* per molecule), suggesting that many nucleosome-free regions are the product of the continual removal of nucleosomes by chromatin remodelers rather than the result of exclusion by DNA sequence [27]. (This does not refute the well-corroborated conjecture that nucleosomes, once formed, preferentially occupy among closely related positions those that are thermodynamically favored [28]. The concepts of nucleosome occupancy and positioning should not be confused.)

It should be noted here that possession of a nucleosome-free or (better) nucleosome-depleted region is not an intrinsic property of promoters, as the classification of promoters into those with and without such a region suggests [29]; the *PHO5* promoter, on average, would be classified as a promoter with and without a nucleosome-depleted region under-activating and repressing conditions, respectively, and single molecules may defy such a classification under any condition [4].

## 6. Transcriptional Bursting

Nucleosomes are general repressors of transcription, in part because they exclude transcription factors from their DNA binding sequences [30]. If single gene molecules visit alternative nucleosome configurations—some conducive to transcription and others not—in random sequence and with statistically distributed sojourn times in between transitions, then transcription is predicted to occur in stochastic bursts.

**Figure 2:**
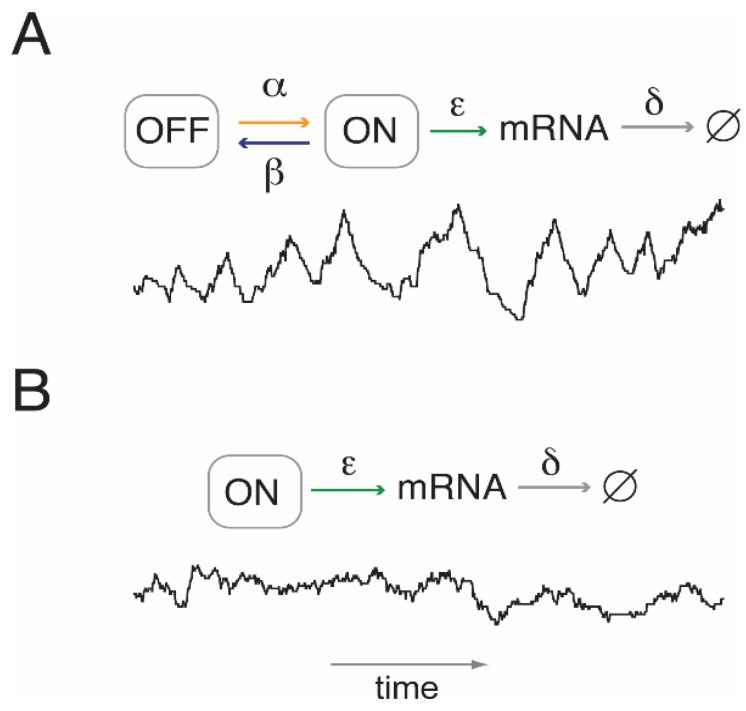
Transcript number fluctuations with and without transcriptional bursting. (**A**) An example or “sample path” (black line) for transcription with random transitioning between two promoter states, ON and OFF, that are conducive and inconducive to transcription, respectively. The sample path was calculated using Gillespie’s stochastic simulation algorithm [33]. The level of expression may be controlled by either one of four parameters: burst frequency, α; burst duration, β^−1^; the initiation rate of transcription in the ON state, ε; and the rate per molecule of mRNA degradation, δ. (**B**) Sample path in the absence of transcriptional bursting (random birth-and-death process). The transcript level may be controlled by ε and δ alone.

The simplest stochastic process that may be invoked to model this behavior assumes two promoter states—one conducive to transcription (ON), the other not (OFF)—between which the promoter jumps randomly, *i.e.*, with statistically distributed sojourn times between jumps (Figure 2) [31,32]. As indicated above, ON and OFF states may each consist of multiple “microstates”, e.g., nucleosome configurations [4]. Both the level of model granularity (the number of microstates) and the presence or absence of irreversible transitions between such microstates affect the sojourn time distribution of the ON and OFF state.

This stochastic model of gene expression raises interesting new problems. On the assumption of a stationary Markov process with two promoter states, the mean number of mRNA molecules or “level of transcription”, *M_R_*, is given by

$$M_{R}=\frac{\text{ε}}{\text{δ}}\frac{\text{α}}{\text{α}+\text{β}}$$

where α and β are the kinetic parameters for the transition from OFF to ON, and ON to OFF, respectively; ε and δ are the parameters for mRNA synthesis (in the ON state) and degradation, respectively [32].

In the absence of bursting (*i.e*., β = 0), the transcript level may be regulated either by controlling the kinetic parameter for mRNA synthesis, ε, or transcript stability, δ. Bursting, furthermore, allows for regulation at the level of burst frequency, α, and burst duration, β^−1^.

By which of these mechanisms do transcriptional activators control transcription [34]? As detailed below, analysis of this problem, while providing novel insights into the mechanism of transcriptional regulation, also suggested experimental tests of the bursting conjecture and, by implication, of the stochastic process theory of promoter nucleosome dynamics.

## 7. Noise and the Mechanism of Transcriptional Regulation

**Figure 3:**
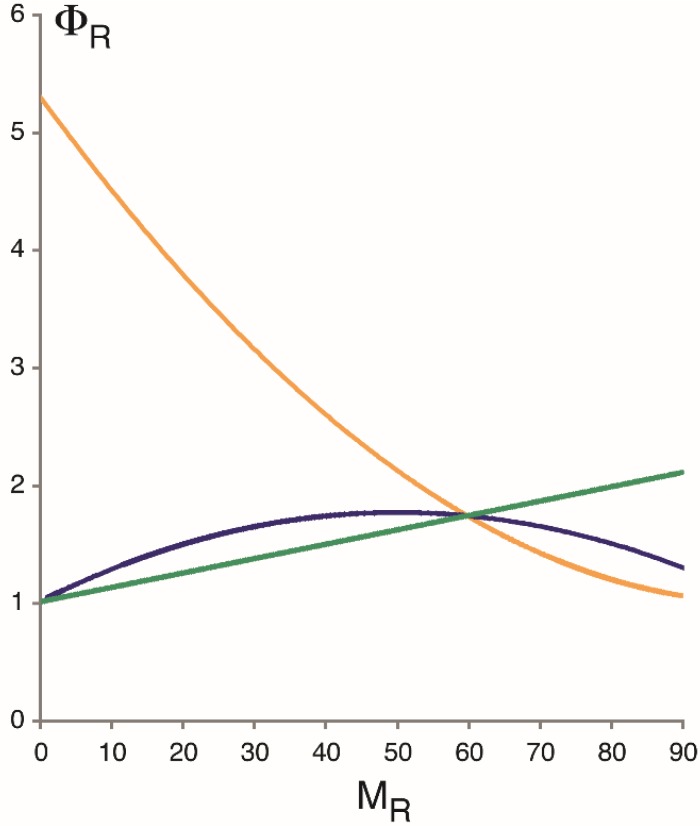
Fano factor for RNA expression, Φ*_R_*, as a function of average transcript number, *M_R_*. (Likewise, the ‘unit’ of Φ*_R_* is number of mRNA molecules.) Curves were calculated on the assumption of a stationary Markov process with two promoter states (Figure 2A), with transcription controlled either by tuning α (burst frequency, yellow curve), β^−1^ (burst duration, blue curve), or ε (the initiation rate of transcription in the ON state, green curve). See main text for equations. The parameters for *M_R_* = 60 are ε = 6.75 per minute, α = 2.18 per minute, β = 1.5 per minute, and δ = 0.066 per minute and molecule [4]. In the absence of transcriptional bursting, Φ*_R_* invariably equals 1.

Stochastic bursting contributes to the variation in the number of mRNA molecules or “noise” of gene expression. The magnitude of this noise may be expressed by the Fano factor, Φ*_R_*, the variance over the mean number of transcript molecules. For the stationary Markov process with two promoter states, the Fano factor is given by

$${\text{Φ}}_{R}=1+\frac{\text{εβ}}{\text{κ}(\text{κ}+\text{δ})}$$

where κ = α + β [32]. In the absence of bursting, Φ*_R_* invariably equals 1, regardless of expression level. Deviations from this expectation may be explained on the conjecture of transcriptional bursting. Thus, Φ*_R_* increases monotonically with increasing mean (*M_R_*) if transcription is controlled by tuning ε (Figure 3; green line) but decreases if regulated via α (yellow line). If regulation occurs at the level of burst duration, then Φ*_R_* describes a convex arch when plotted as a function of *M_R_* (blue line). The same qualitative conclusions hold for protein expression, and for promoter models with several ON and OFF states [4]. The different regulatory mechanisms may be tested, therefore, by measuring the mRNA or protein noise at different levels of expression of the same gene.

To test regulatory hypotheses, two noise components must be distinguished, which together comprise the total noise of gene expression: “extrinsic noise”, which results from the deterministic response of the gene expression process to variation in its intracellular environment (extrinsic variation), and “intrinsic noise”, which cannot thus be explained and must arise, therefore, within the gene expression process and not elsewhere [35].

Experimentally, the noise components can be determined by employing the conjugate reporter approach: two gene copies that encode distinguishable gene products, but are otherwise identical, are observed within the same cell [35,36]. The deterministic response to extrinsic variation induces a correlation between the conjugate reporters from which the extrinsic noise is calculated. The remaining noise is intrinsic noise. That extrinsic noise arises from a deterministic response to extrinsic variation may be seen by considering gene expression in the absence of intrinsic noise. In this instance, both conjugate reporter genes would be perfectly correlated; the expression state of one reporter is completely determined by the expression state of the other reporter via their common environment, and hence, by the environment. It may be said, therefore, that extrinsic noise is “informative variation” [36].

**Figure 4:**
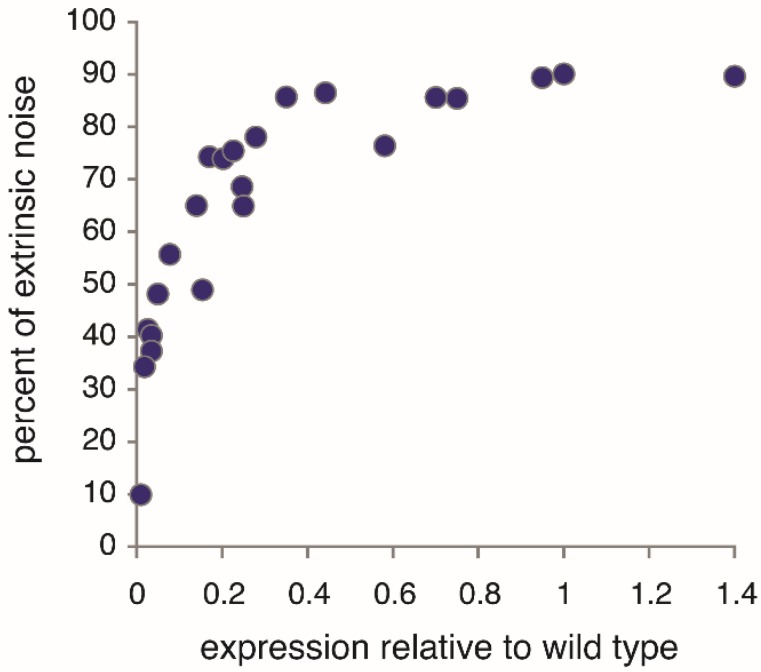
Extrinsic protein noise of *PHO5* promoter-driven gene expression as percentage of total noise at different levels of *PHO5* expression. Under fully activating conditions (relative expression of 1), extrinsic noise is the dominant noise component. Noise components were determined by the conjugate reporter approach (see main text) with CFP and YFP (derivatives of the green fluorescence protein or “GFP”) expressed under control of the *PHO5* promoter [7,37]. (These data have not been previously published.) *PHO5* expression was analyzed in *pho80*Δ cells, which express *PHO5* constitutively, and manipulated by mutating the activation domain of *PHO*4, the transcriptional activator of *PHO5* [38], or by *PHO5* mutation in the binding sequences of *PHO*4 [7]. Protein expression was normalized by cell size, a significant contributor to extrinsic variation. Without this normalization, the extrinsic noise was close to 97% of the total noise for the wild type, consistent with earlier measurements [37].

Under fully activating conditions, extrinsic noise is the dominant noise component of *PHO5* expression; *i.e.*, most variation in *PHO5* expression is explained by extrinsic variation (Figure 4). Surprisingly, the opposite is the case for the structural variation of promoter chromatin. The nucleosome configurations of two *PHO5* promoter copies were found to be stochastically independent [11]; that is to say, the variation in nucleosome configuration could not be explained whatever by extrinsic variation. It had to entirely arise “intrinsically”. The resulting noise in gene expression, then, is intrinsic noise. This was naively assumed previously [4,5,7], without experimental testing. The assumption was bold, for an effect of extrinsic variation on the promoter nucleosome configuration appeared likely, given it is the dominant influence on the noise of *PHO5* expression (Figure 4).

Measurements of the intrinsic noise at different levels of *PHO5* protein expression (but not extrinsic or total noise) indicated an increasing Fano factor with decreasing expression level [4,7]; that is to say, the measurements fit the predictions of burst frequency control and not other regulatory conjectures (Figure 3), thus corroborating, by implication, the bursting hypothesis. Regulation of transcription by burst frequency is not limited to *PHO5* [39].

## 8. Accelerated Removal *versus* Exclusion

Burst frequency control has an important implication: the net loss of promoter nucleosomes observed upon transcriptional induction of *PHO5* must be due to accelerated disassembly rather than slowed reformation of nucleosomes—the first increases burst frequency, the latter burst duration. The hypothesis that loss of nucleosomes at the *PHO5* promoter is due to steric exclusion by promoter-bound transcription factors, *i.e.*, decelerated nucleosome formation, was independently refuted [4]. Thus, nucleosomes exclude transcription factors from their DNA binding sites, and not *vice versa*. In this context, it is of great interest that the SWI/SNF remodeling complex can dislodge transcription factors from DNA by catalysis of nucleosome sliding [40]. This may explain why the dwell time of transcription factors at their binding sites *in vivo* is markedly shorter than their dwell time on naked DNA *in vitro* [41].

Consistently, it is widely believed that activators stimulate transcription, among other mechanisms, by the recruitment of chromatin remodelers to promoters to relieve nucleosomal repression—in other words, by accelerating nucleosome removal. Chromatin immunoprecipitation experiments have supported this conjecture [42]. However, such experiments neither revealed the fate of nucleosomes, nor answered the question of whether nucleosome loss was attained by exclusion or accelerated removal; the theoretical and experimental analysis of molecular variation did because it enabled the decision between competing conjectures of cause and effect.

Because nucleosomes may form on any DNA sequence, albeit with different free energies [43], the conjecture of transcriptional stimulation by accelerated nucleosome removal predicts, together with the stochastic process theory of promoter chromatin, that all (eukaryotic) genes are regulated, at least in part, at the level of burst frequency. However, no such consensus has been reached across different studies. Some transgenes were found to be regulated by burst size rather than burst frequency [44]; and transcription of other genes did not appear to occur in bursts at all [45].

It has been argued that most promoter mutations affect the frequency rather than the size of transcriptional bursting [29]. However, this conclusion was based on the fitting of total (and not intrinsic) protein noise data to a Markovian model of gene expression which assumes that promoters are continually transcribed; that transcription does not occur in bursts [46,47]. Thus, the size of translational bursts (the average number of protein molecules translated per mRNA) was inferred from the data, and not transcriptional bursts. This may explain, perhaps, why most promoter mutations did not affect burst size, and those that did frequently generated upstream start codons [29]. The same approach was used to interpret the effects of loss-of-function mutations in genes for histone-modifying enzymes on total protein noise [48]. The implications of the reported data from these studies for transcriptional bursting are uncertain. To the extent that the applied model fit the data, the hypothesis of transcriptional bursting was not required to explain them.

## 9. Further Analysis is Warranted

Transcriptional bursting, the theory that promoters randomly transition between ON and OFF states, is an explanatory conjecture. It has been invoked to explain, essentially, two kinds of observations: a variable Fano factor with changing expression level (Figure 3), and the appearance of short and long gaps between transcription initiation events [4,7,37,39,44,49]. It should be kept in mind that the same observations may perhaps be explained otherwise. Explanatory theories can be tested, but not observed. (Explanations necessarily transcend the observations they explain.) Further testing is warranted: the above discussion shows that the notion of transcriptional bursting is closely linked to hypotheses of transcriptional regulation. This means that tests of the bursting hypothesis are essentially tests of regulatory conjectures. To this end, expression noise should be analyzed at different levels of expression of the same gene. Intrinsic and extrinsic noise must be experimentally distinguished (by employing the conjugate reporter approach), for pulsatile transcription may arise from both extrinsic and intrinsic variation*.* Genes that are strongly expressed naturally (such as *PHO5*) provide ideal models because their expression level may be manipulated over several orders of magnitude: for instance, by shortening the activation domain of their transcriptional activator, which addresses the question of how transcription is controlled physiologically [7], *viz*. by activators rather than chromosomal location [49,50].

Recent developments have made it possible to monitor nascent transcription at single gene loci in life cells over time [39,45]. This should allow for addressing the critical question of whether transcription occurs in bursts in real time, in instances where it previously had been inferred from population variation; in other words, the ergodic hypothesis may thus be tested. Temporal observations also provide the means to test the proper granularity of promoter models, *i.e.*, the number of promoter states required to explain noise. Thus, deviations from an exponential sojourn time distribution have hinted at two or more OFF states [51,52]. Consistently, it has been argued that several nucleosome configurations of the *PHO5* promoter are inconducive to transcription [4]. This predicts a refractory period prior to the reactivation of transcription, as the promoter passes through two or more OFF states before reentering an ON state.

In principle, it is possible to determine promoter model granularity by recording the autocorrelation function for RNA fluctuations in single cells, which indicates the correlation between two RNA measurements at time points *t*_1_ ≤ *t*_2_ as a function of τ = *t*_2_ – *t*_1_. The autocorrelation function decreases monotonically from one to zero with increasing τ, as the process “forgets” its increasingly distant past. Provided certain mathematical conditions (*viz*. that transcription is a stationary Markov process with a diagonalizable generator for promoter transitions), it can be proved that the autocorrelation function for an *n*-promoter state model is the sum of *n* exponential functions (H. Boeger, unpublished; but for solution of the analogous problem for transmembrane ion channels see [53]). However, the temporal resolution of current methods is likely insufficient to fully attain this goal; further technological advances are required.

Similar to Fano factor analysis, different regulatory modes of transcriptional bursts may be tested by autocorrelation analysis; different modes predict characteristic shifts in the autocorrelation time with changes in mean expression. Thus, if transcription occurs in bursts and is regulated by burst frequency, the autocorrelation function shifts toward longer times with decreasing mean expression, but it shifts toward shorter times if regulated by burst size. No shift is expected if transcription is controlled by the rate of RNA synthesis without bursting. This analysis may reveal bursting where it previously was not observed because autocorrelation measurements were conducted at one level of expression only. (Transcriptional bursting is masked if the promoter dynamics are significantly faster than the rate of RNA synthesis, if ε << κ; see equation for Φ*_R_* above.)

Other critical tests of the bursting hypothesis are tests of its conjectured molecular basis, which is unknown. (For this reason alone, it should be clear that bursting is not observed, but hypothesized.) The random process theory of promoter chromatin expounded here provides such a conjecture, *i.e.*, a molecular explanation for random bursting. It currently is the most detailed and best-tested explanation, but other mechanisms likely exist: the observed range in nucleosome occupancy did not explain the observed range in *PHO5* transcription, suggesting that the frequency of bursting is not controlled at the level of promoter nucleosomes alone [4]. Furthermore, transgenes in mammalian cells appear to burst at a different time scale than genes in Drosophila and yeast [4,39,44,51,54], suggesting different underlying mechanisms. Noise measurements in mammalian cells may be affected by the propensity of transgenes to fall victim to gene silencing. The mechanism remains obscure, but chromatin appears to play a role [55]. The chromosomal integration site of transgenes has been found to affect both burst size and burst frequency [49,50,56]. The molecular basis of such locus effects is unknown. Again, chromatin structure may play a role [49].

## 10. Concluding Remarks

The analysis of both promoter chromatin structure and gene expression at the level of single gene molecules has indicated a surprising degree of intrinsically random behavior. Both appear mechanistically linked: the intrinsically stochastic dynamics of promoter chromatin provide an explanation for intrinsically stochastic bursting in transcription. Other, additional mechanisms for bursting likely exist. The regulation of transcription by control of burst frequency or duration is unique to the probabilistic theory of gene expression; gene regulation may not be understood in deterministic terms. However, the deterministic response of genes to environmental signals is an apparent requirement for biological function in many instances, such as embryonic development [54,57]. How is this determinism reconciled with the intrinsically random behavior of single gene molecules? This problem—of chance and necessity [58], of clouds and clocks, as it were [59]—is not new; it has been pondered by philosophers and biologists alike. Darwin’s stroke of genius explained the emergence of purposive or teleonomic properties from random variation by selection. Randomness in gene expression, perhaps, may similarly be understood as a prerequisite for biological function rather than its impediment.
